# Association of Initial Muscle Fitness with Weight Loss and Metabolically Healthy Status in Children and Adolescents with Obesity: A Retrospective Study

**DOI:** 10.3390/metabo14090468

**Published:** 2024-08-24

**Authors:** Wenya Shang, Jiaqi Kong, Mengxue Zhang, Tao Chen, Linlin Zhao, Kun Wang, Qin Yang

**Affiliations:** 1 School of Rehabilitation Medicine, Henan University of Chinese Medicine, Zhengzhou 450046, China; shangwenya435@gmail.com; 2 International College of Football, Tongji University, Shanghai 200092, China; 2333822@tongji.edu.cn (J.K.); 2333825@tongji.edu.cn (M.Z.); 3 Department of Physical Education, Tongji University, Shanghai 200092, China; chentwhy@tongji.edu.cn; 4 School of Physical Education, Shanghai Normal University, Shanghai 200234, China; zll0907@shnu.edu.cn; 5 Shanghai Dianfeng Sports Management Co., Ltd., Shanghai 200441, China; yangqin19860704@gmail.com

**Keywords:** muscle fitness, weight loss, metabolically healthy obesity, children and adolescents, cardiovascular risk

## Abstract

This retrospective study aimed to investigate the association of initial muscular fitness (MF) with weight loss and metabolic health status in 282 children and adolescents with obesity during 3 to 4 weeks of diet- and exercise-based interventions. Metabolically healthy obesity (MHO) definitions established in 2023 and MF standards based on the 2021 Chinese children’s grip strength grading were applied. The proportion of metabolically unhealthy obesity (MUO) was higher in the high MF group than in their low MF counterparts at baseline. After the intervention, neither group transitioned from MUO to MHO due to the high frequency of low HDL-C. High MF females showed a higher percentage of high systolic blood pressure (SBP) than low MF females before and after intervention. High MF males exhibited greater improvements in waist circumference, hip circumference, waist-hip ratio, triglycerides, total cholesterol, and LDL-C than low MF males. The benefits of weight loss and blood lipids obtained by males are more evident than those obtained by females under the same MF level. Thus, attention should be paid to females during weight loss regardless of MF levels. Precision therapy should prioritize the management of blood pressure and avoid excessive reduction in HDL-C levels to sustain metabolic health.

## 1. Introduction

Childhood obesity is a chronic metabolic disease influenced by numerous factors and is a public health concern worldwide. The World Obesity Federation estimated that 254 million children will grapple with obesity by 2030 [1]. The 2020 Report on Nutrition and Chronic Diseases Status of Chinese Residents reveals that 19% of children (aged 6–17 years) and 10.4% of those under 6 years of age are currently overweight and obese [2]. This highlights a concerning situation regarding the prevention and management of childhood overweight and obesity, which is likely to persist into adulthood, correlating with cardiovascular disease, metabolic disorders, and premature mortality [3].

In the 1980s, researchers introduced the term “benign obesity phenotype”, describing a type of obesity without hypertension, cardiovascular disease, insulin resistance, diabetes mellitus, dyslipidemia, or metabolic syndrome. According to their findings, the concept of metabolically healthy obesity (MHO) has emerged [4]. Research on adults has demonstrated that individuals classified as MHO comprise approximately 30% of all individuals with obesity, and they exhibit a lower risk of cardiovascular disease compared to those classified as metabolically unhealthy obese (MUO) [5,6]. Nevertheless, the MHO and MUO categories gained comparable weight loss benefits in children and adolescents with obesity. However, the concept of MHO in children was introduced only recently in 2018, and scholars still unanimously acknowledge its criteria for determination [7]. Research on MHO in children is still in its infancy. Few studies have cconcentrated on changes in metabolic health status and sex differences in preventing and treating obesity among this population.

The term “muscle fitness (MF)” refers to muscular strength, local muscular endurance, and muscular power [8]. Low levels of MF are considered crucial markers of nutritional status and predictors of metabolic disease, cardiovascular disease, and all-cause mortality [9,10,11]. Research indicates that MF may be negatively associated with early cardiometabolic risk factors, manifested by a reduction in the prevalence of some cardiovascular diseases by approximately 15–20% through an increase in grip strength by 5 kg [12,13]. This phenomenon may indicate sex disparity. Pratt et al. [14] observed stronger associations between grip strength and cardiorespiratory fitness in females than in males. Celis-Morales et al. [15] observed hazard ratios for all-cause mortality of 1.16 in men and 1.20 in women per 5 kg decrease in grip strength. Furthermore, reduced MF in young individuals is associated with an increased risk of cardiovascular disease and mortality in adulthood, independent of their body mass index and cardiorespiratory fitness.

Multicomponent lifestyle interventions, including dietary modification and structured physical activity, are sufficient to achieve notable health benefits in anthropometry and cardiometabolism among children and adolescents with obesity [16]. Nonetheless, varying the initial states of subjects, such as initial metabolic health status, may yield different weight loss outcomes. In adolescents with obesity, maintaining optimal MF levels can retain skeletal muscle structure and function and reduce the levels of inflammatory factors [8]. However, it remains unclear whether the initial level of MF influences weight loss and metabolic health status. This retrospective study aimed to investigate the association of initial MF with weight loss and metabolic health status in 282 children and adolescents with obesity during 3 to 4 weeks of diet- and exercise-based interventions. These results will contribute to the development of personalized interventions for children and adolescents with obesity from various perspectives, thus preceding the management of childhood obesity.

## 2. Materials and Methods

### 2.1. Study Population and Database

This retrospective study recruited 1063 children and adolescents with obesity who participated in the Weight Loss Camp organized by the Shanghai Dian Feng Weight Loss Center between 2017 and 2019. Notably, the camp excluded subjects treated with bariatric medications or surgery, and those with hepatic, renal, or cardiac diseases. Based on the data extracted from the weight loss camp, we carried out further screening. Subjects whose ages were ≤6 years or ≥18 years (*n* = 37) and those who attended the camp for less than 3 weeks or more than 4 weeks (*n* = 105) were excluded. Individuals who missed the initial or post-intervention anthropometric and metabolic parameters (*n* = 639) were also excluded from the study. Finally, 282 children and adolescents with obesity from camps, consisting of 151 males and 131 females, were included in this study. Participants and their parents provided informed consent following a detailed explanation of the research. This retrospective study was conducted and reported in adherence to the STROBE guidelines (http://www.plosmedicine.org/, accessed on 16 October 2007). The research adhered to the tenets of the Declaration of Helsinki and obtained approval from the Ethics Committee at Tongji University (No. 2021tjdx046; 9 March 2021). The research outcomes remained uninfluenced by any related company.

### 2.2. Diet and Exercise Protocol

This retrospective study included the most representative intervention period of 3–4 weeks to minimize the specific effects of the intervention periods on the outcomes. All subjects received a professional and individualized lifestyle intervention for an average duration of 24.5 ± 3.2 days. Daily energy expenditure was evaluated according to the measured basal metabolic rate and treadmill test [17]. The prescribed diet was designed by professional nutritionists to restrict total caloric intake, comprising high-quality protein (15–20%) to maintain nitrogen balance during exercise, carbohydrates (50–60%), with strict control over fat intake (15–20%), and ensuring a fiber intake of 8.5 g/1000 kcal [18]. According to the Dietary Guidelines for Chinese Residents (2016) [19], the primary recommended food categories include vegetables (spinach, kale, celery, etc.), fruits (banana, apple, pitaya, etc.), legumes, eggs, whole grains, nuts, fish, poultry, and low-fat dairy products. Olive oil was recommended as the main culinary method, and vitamin and mineral supplements including potassium chloride, calcium carbonate, vitamin K, sodium, and magnesium were provided to the subjects. Processed foods, bakery, sugars, and sugary drinks were discouraged. Furthermore, booklets on food calorie content and health lectures were provided to the participants and their parents to help them develop healthy lifestyle habits. No specific training was conducted to enhance the muscular fitness of the participants.

An incremental treadmill test was performed using a flat treadmill (H/P/Cosmos Pulsar, Nussdorf-Traunstein, Germany) to determine exercise intensity. Under the presumption of a normal resting 12-lead electrocardiogram (Nippon Kohden Kogyo Co., Ltd., Tokyo, Japan, ECG-9130P), participants began running at a speed of 4 km/h, increased by 2 km/h every 2 min, then paused for 10 s to record the immediate electrocardiogram (ECG), and stopped the test at a speed of 8 km/h [20]. When deciding on exercise intensity, we must consider how to effectively burn fat and avoid exercise-related injuries. To facilitate weight loss, the target heart rate was computed as the resting heart rate (RHR) plus (maximum heart rate − RHR) × (20% to 40%) [21]. A finger-clip pulse oximeter (NONIN Onyx^®^II, Plymouth, MN, USA, Model 9550) was used to monitor the heart rate. Exercise intensity was adjusted based on whether the heart rate was within the target range. Considering the severity of obesity and the pace of weight loss, an exercise program with 2 sessions a day and 6 days per week was implemented. To avoid muscle strain, each session should include 15 min of warm-up, 5 to 10 min of relaxation, and a 5 min break after every 30 min of exercise (There are usually three cycles of “30 min + 5 min”). The exercise types were selected based on personal interests and suitability for each individual, ensuring moderate intensity and reduced stress on the ankle and knee joints. Generally, the exercise program consisted of brisk walking, aerobics, swimming, cycling, fun ball games, etc. In addition, the subjects were provided with sports drinks containing sodium, potassium, and calcium.

### 2.3. Data Collection

The methods for obtaining data in this study are outlined as follows: Questionnaires were used to collect information on demographic characteristics (age, sex, medical history, and lifestyle) [22]. Grip strength was measured by an electronic grip strength meter (EH101; Zhongshan, China). The subject stood with arms hanging down naturally on both sides of the body, ensuring that the shoulder joints were tucked in, while the dominant hand squeezed the handles with maximum force for at least 5 s. Measurements were performed two times, with an interval of at least 30 s, and the maximum of the two measurements was recorded as the maximal grip strength. Body weight (BW) and height were measured using a digital scale (Yaohua Weighing System Co., Shanghai, China), and body fat ratio (BFR) was measured using a body composition analyzer (TANITA TBF-418B, Tokyo, Japan). BW was assessed with participants wearing underwear, while height was assessed with participants not wearing slippers. The Grip Strength Index was calculated as the maximal grip strength divided by weight in kilograms. Body mass index (BMI) was calculated as the weight in kilograms divided by the square of the height in meters (kg/m^2^). Circumference was measured without clothing at least 2 times (take the average value if the error between the two measurements was less than 1 cm) by the same technician using a measuring tape. Chest circumference (CC) was measured at nipple level, waist circumference (WC) was measured above the uppermost lateral border of the right iliac crest, and hip circumference (HC) was measured as the distance around the widest part of the buttocks. CC, WC, and HC were measured in participants under natural and relaxed conditions. Systolic blood pressure (SBP) and diastolic blood pressure (DBP) were measured using a sphygmomanometer (Nishimoto Sangyo Co., Tokyo, Japan). Heart rate was monitored via a 12-lead electrocardiogram and a polar heart rate telemetry device. For blood indices, 5 mL of twelve-hour overnight fasting blood samples were extracted from the ipsilateral elbow vein. It was left at room temperature for 40 min and centrifuged for 15 min to extract the supernatant serum, which was then sent to the Adicon Medical Laboratory Centre (Shanghai, China, a medical testing company that has obtained “China Inspection Body and Laboratory Mandatory Approval” certification) to assess fasting blood glucose (FBG), triglyceride (TG), total cholesterol (TC), high-density lipoprotein–cholesterol (HDL-C), and low-density lipoprotein–cholesterol (LDL-C) levels. All assessments were performed by trained assessors from the Edicon Company during the research.

### 2.4. Definition of High-MF and Low-MF

In 2021, Chinese scholar Ji Liu applied the LMS method to establish theoretical cut-off points for the four-level scores of each physical fitness indicator in Chinese children. It is aligned with the percentile method’s classical evaluation level division standard [23]. The evaluation indicators were categorized as fail (P < 10th percentile), pass (10th percentile ≤ P < 75th percentile), good (75th percentile ≤ P < 90th percentile), or excellent (P ≥ 90th percentile). Based on the physical health standards for Chinese students, this retrospective study classified individuals with grades in the “fail” and “pass” ranges into the low MF group, while those in the “good” and “excellent” ranges were classified into the high MF group.

### 2.5. Definition of MHO and MUO

The diagnostic criteria for MHO established in 2023 were applied as follows: (1) HDL-C levels of >1.03 mmol/L (or >40 mg/dL); (2) TG levels of ≤1.7 mmol/L (or ≤150 mg/dL); (3) SBP or DBP levels ≤90th percentile; and (4) FBG levels ≤5.6 mmol/L (or ≤100 mg/dL) [7]. Subjects who did not meet any of the four criteria were classified as having MUO. The 2018 Chinese industry standards for children were used to identify central obesity (defined as the 95th percentile for age and sex) and abnormal blood pressure (BP) defined as the 90th percentile for age, sex, and height) [24,25].

### 2.6. Statistical Analyses

All data were analyzed using SPSS 25.0, and descriptive data are presented as mean ± standard deviation (SD). The Kolmogorov-Smirnov test was conducted to test the normality of the variables. When comparing the baseline data of different groups, the independent samples *t*-test and chi-square test were used to analyze continuous and categorical variables, respectively. Changes in anthropometric and metabolic indicators before and after the intervention within each group were analyzed using paired *t*-tests. To compare post-intervention indicators between the low and high MF groups in terms of variables, we used analysis of covariance (ANCOVA) with initial MF status as the between-subjects factor and the baseline value of the analyzed indicators, age, height, and days of intervention as covariates. Significance was determined at a 2-tailed *p*-value < 0.05.

## 3. Results

### 3.1. Basic Characteristics of the Two Groups

A total of 282 children and adolescents with obesity (mean age, 12.9 ± 2.3 y; 151 males) were included and grouped by MF status in this retrospective study. Overall, 188 (100 males) and 94 (51 males) children and adolescents with obesity were categorized into the low MF group and high MF group at baseline, respectively. Except for height (*p* = 0.049), there were no significant differences in anthropometric and blood indicators between the two groups (all *p* > 0.05) (Table 1).

### 3.2. Distribution of Indicators Related to Metabolically Healthy Status in the Low MF and High MF Groups before and after Intervention

We first compared the metabolic health indicators at different MF levels before and after the intervention. Table 2 shows that before the intervention, more individuals in the high MF group (72.3%) exhibited MUO compared to those in the low MF group (59.6%) (χ^2^ = 4.424, *p* < 0.05). The most frequent metabolically healthy risk factor in the low and high MF groups was high SBP (37.2% and 50%, respectively), followed by high DBP (>the 90th percentile for age, sex, and height) (28.7% and 36.2%) and low HDL-C (<1.03 mmol/L) (23.9% and 33%). After the intervention, neither group transitioned from MUO to MHO, according to the chi-square test results (all *p* > 0.05). The most frequent metabolically healthy risk factor shifted to low HDL-C levels in all subgroups. Additionally, we identified individuals who were initially in MHO status but exhibited abnormal changes in SBP, DBP, or FBG indicators after the intervention. Except for HDL-C (χ^2^ = 7.257, *p* < 0.01), no significant differences were observed in the changes in other metabolically healthy indicators after intervention between the low MF and high MF groups.

### 3.3. Distribution of Indicators Related to Metabolically Healthy Status in the Low MF and High MF Groups among Different Sex before and after Intervention

Next, we compared variations in metabolic health indicators before and after the intervention among males and females with different MF levels. Table 3 reveals no significant sex difference between the low and high MF groups before the intervention (χ^2^ = 0.029, *p* > 0.05). The most frequent metabolically healthy risk factor in males in the low and high MF groups was high SBP (22.3% and 26.6%, respectively), followed by low HDL-C (16% and 19.1%) and high DBP (15.4% and 16%). Similar to males, the most frequent metabolically healthy risk factors in low MF and high MF females were high SBP (14.9% and 23.4%, respectively), followed by high DBP (13.3% and 20.2%) and low HDL-C (8% and 13.8%). After the intervention, low HDL-C emerged as a predominant metabolically healthy risk factor in males (29.3% and 31.9%) and females (15.4% and 18.1%) in the low and high MF groups. Furthermore, females in the high MF group were still exhibiting high SBP (14.9%) after the intervention. Even when performing an overall analysis of the low MF and high MF groups without sex differentiation, we obtained similar results.

### 3.4. Changes of Anthropometry and Blood Indicators in Low MF and High MF Groups before and after Intervention

Finally, we compared changes in anthropometry and cardiometabolic indicators between both sexes in the low MF and high MF groups before and after intervention. According to the classification criteria for MF, Table 4 shows that before the intervention, the mean BFR (*p* < 0.05) in the high MF males was significantly lower than that in the low MF males, while grip strength and grip strength index were significantly higher in the high MF males than in the low MF males (all *p* < 0.01). In contrast, high MF females exhibited significantly higher BW (*p* < 0.01), BMI (*p* < 0.01), grip strength (*p* < 0.01), grip strength index (*p* < 0.01), CC (*p* < 0.05), WC (*p* < 0.01), HC (*p* < 0.05), and SBP (*p* < 0.05) compared to low MF females. Additionally, high MF females exhibited significantly higher BFR (*p* < 0.01), while grip index (*p* < 0.05) and WHR (*p* < 0.01) were significantly lower than those of high MF males. Low MF females exhibited significantly lower BW, BMI, grip strength, CC, WC, WHR, and SBP than low MF males (all *p* < 0.01). Overall, after 3 to 4 weeks of diet- and exercise-based interventions, the decreased rates of BW for low MF males, low MF females, high MF males, and high MF females were (9.75 ± 1.68) × 100%, (10.07 ± 2.18) × 100%, (9.75 ± 1.70) × 100%, and (9.50 ± 2.11) × 100%, respectively.

After intervention, most indicators in subgroups demonstrated significant improvement (*p* < 0.05 or *p* < 0.01), except for FBG of low MF males and FBG and grip strength of high MF females. Except for the high MF females, changes in both grip strength and grip strength index were consistent across the remaining three groups. Although grip strength did not show a significant improvement in high MF females (*p* > 0.05), BW decreased significantly (*p* < 0.01). Consequently, the grip strength index (the ratio of grip strength to BW) exhibited a significant change (*p* < 0.01). It is worth noting that grip strength in the high MF males (*p* < 0.05) and HDL-C in all four subgroups showed a significant decrease (*p* < 0.05 or *p* < 0.01) after intervention.

After the intervention, the change levels in WC (*p* < 0.01), HC (*p* < 0.05), WHR (*p* < 0.05), TG (*p* < 0.05), TC (*p* < 0.01), and LDL-C (*p* < 0.05) were significantly higher in high MF males than in low MF males. Furthermore, low MF females exhibited smaller changes in BW (*p* < 0.01), BMI (*p* < 0.01), WHR (*p* < 0.05), TC (*p* < 0.01), HDL-C (*p* < 0.01), and LDL-C (*p* < 0.05) compared to low MF males. High MF females exhibited smaller changes in BW (*p* < 0.05), RHR (*p* < 0.01), TG (*p* < 0.01), TC (*p* < 0.01), HDL-C (*p* < 0.01), and LDL-C (*p* < 0.01) compared to high MF males.

## 4. Discussion

To our knowledge, this is the first study to investigate variations in anthropometry and cardiometabolic changes among male and female children and adolescents with obesity at different MF levels. According to the classification criteria for MF, high MF children and adolescents with obesity had a higher prevalence of metabolic health issues than their low MF counterparts. The most prevalent metabolic health issues at different MF levels were high BP and low HDL-C levels, regardless of sex. A heightened percentage of individuals with low HDL-C levels after the intervention impeded the subgroups from maintaining or transitioning to MHO status. Both male and female individuals with obesity showed significant differences in anthropometry and cardiometabolic indicators under different MF levels at baseline. BFR was lower in high MF males than in low MF males, while BW, BMI, CC, WC, HC, and SBP were higher in high MF females than in low MF females. The diet- and exercise-based intervention significantly improved most anthropometric and cardiometabolic indicators in all groups except HDL-C (in all subgroups) and FBG (in low MF males and high MF females). Nevertheless, variations in the extent of metabolic health improvement were observed between males and females with obesity at different MF levels. This study revealed that males with high MF experienced greater improvements in body circumference and blood lipids from the diet- and exercise-based lifestyle intervention, while this phenomenon was not observed in females. Additionally, it is imperative to exercise caution to prevent a decline in HDL-C levels and elevated SBP during weight loss, thereby preserving the metabolic health status of the organism.

MF can be measured through various strength performance tests, including assessments of grip strength, explosive lower body strength (jumping), and muscular endurance (sit-ups) [26]. Grip strength is primarily measured using a straightforward fixed dynamometer. The testing procedure is convenient, safe, and independent of location. Moreover, it mitigates the challenge of low discriminative utility linked to the incomplete execution of diverse upper limb strength testing protocols. Therefore, this study utilized reliable and effective grip strength testing to evaluate MF.

The absence of a standardized definition has led to diverse thresholds for MHO diagnosis, resulting in a significant variation in MHO prevalence across studies. The prevalence of MHO among individuals with obesity is also influenced by factors such as sex, age, ethnicity, region, and lifestyle [27]. Rey-López et al. [28] reported a global range of MHO prevalence among adults with obesity ranging from 6% to 75%, with a notable decrease with increasing age. Furthermore, they observed a higher prevalence of MHO in females compared to males. In a study involving Chinese adults, MHO prevalence ranged from 4.2% to 13.6%, while a study of African Americans reported a higher MHO prevalence of 28.5%, indicating notable geographical variation [29]. The prevalence of MHO in our study (28–40%) aligns with that reported by Genovesi et al. [30] (20–68%), but surpasses another study conducted by Chen et al. [31] (15.3%). Dundar et al. [32] reported a consistent prevalence of MHO in boys and girls, approximately 34%. In contrast, Cai et al. [33] observed a sex disparity in MHO prevalence among Chinese children, with boys exhibiting a higher prevalence of MHO than girls. Our prior study confirmed the findings of Dundar et al. that the prevalence of MHO remains consistent in adolescents with obesity of both sexes [6]. In line with our previous report, this observation may be related to alterations in sex and growth hormone secretion during puberty.

This study identified variations in the percentage of MUO and metabolically healthy risk factors between males and females, depending on the discrepancy in initial MF levels. There was a higher percentage of metabolic health issues in children and adolescents with obesity among individuals with high MF. Without sex distinction, the most prevalent metabolically healthy risk factors in both the low and high MF groups included high SBP, high DBP, and low HDL-C. However, upon distinguishing between sexes, the frequency of low HDL-C was higher than that of high DBP in males. Two previous studies identified high SBP and high TG levels as the most prevalent risk factors for MUO in children [30,34]. Variations in the MUO definition may explain the disparity in the results. In our study, TG level > 150 mg/dL was deemed a risk factor, whereas the above study defined it as TG level ≥ 100 mg/dL (age < 10 years) or ≥130 mg/dL (age ≥ 10 years). Our study employed broader criteria, leading to the finding that high TG levels do not significantly contribute to metabolic health. Furthermore, our study revealed that the diet- and exercise-based intervention did not facilitate the transition from MUO to MHO in any of the groups. We observed that a low HDL-C level was the most prevalent abnormality in all subgroups after intervention. In a multicenter study, participants adhered to an 810 kcal daily dietary regimen for eight consecutive weeks, resulting in weight loss and increased insulin sensitivity. However, it was also observed that the mean HDL-C level decreased to 4.6 mg/dL. Low-calorie diets may lead to a reduction in fat intake, resulting in decreased HDL-C substrate fatty acids and, subsequently, lower HDL-C levels [35]. This phenomenon may explain the observed decrease in HDL-C levels in our study. Potential prevention strategies include increasing the intake of olive oil and nuts within current interventions, along with integrating aerobic exercises like Tai Chi and yoga to enhance the protective effect of HDL-C. Our study found a higher prevalence of low HDL-C in males. Therefore, special attention should be focused on preventing HDL-C decline in male children and adolescents with obesity during weight loss interventions. The observation that MHO individuals showed abnormalities in BP and FBG after intervention indicates evident metabolic plasticity in childhood. Finally, the proportion of high SBP in the high MF-MUO group remained at a high level (16%) after intervention. This could be attributed to the initial high baseline proportion of elevated SBP, which was already as high as 50%. Therefore, special attention should be focused on addressing BP abnormalities in children and adolescents with obesity and high MF, such as implementing additional control over sodium salt intake among individuals with high MF.

There were variations in anthropometric measurements and cardiometabolic indicators between males and females at different MF levels. Before the intervention, BFR was lower in high MF males than in low MF males, whereas BW, BMI, CC, WC, HC, and SBP were higher in high MF females than in low MF females. Kakaraparthi et al. [36] reported a positive correlation between grip strength BW, and BMI in females aged 6 to 12. Consistent with our findings, increased BW, BMI, and body dimensions may contribute to higher MF levels in girls. Interestingly, we found that before the intervention, different levels of MF did not result in significant differences in BFR when sex differences were not considered. However, after accounting for sex differences, high MF levels may be associated with increased muscle mass and function in boys, potentially leading to a lower BFR. This phenomenon was less evident in females. Similarly, Confortin et al. [37] found that the fat mass index was associated with lower grip strength, with this association being more pronounced and nearly twice as strong in males compared to females. This difference may stem from the inherent tendency of girls to possess a higher BFR than boys, which is crucial for maintaining optimal physiological health. Before the intervention, we observed a higher percentage of metabolic health issues among females with high MF than among those with low MF. After the same diet- and exercise-based intervention, we observed a higher percentage of high SBP in females with high MF. Although studies have suggested that adolescents with higher MF levels exhibit lower SBP [38], a positive correlation between grip strength and BP was observed when stratified or adjusted for BMI [39]. For every additional kilogram of grip strength, SBP increased by 0.11 mmHg [40]. Our study focuses on individuals with obesity and supports the latter view. We should exercise caution to address high SBP in females with obesity with high MF during weight loss. Furthermore, in a study involving healthy children, boys demonstrated superior upper limb grip strength compared to girls before puberty. This sex disparity decreased during puberty and increased after puberty [41,42]. However, in our study involving children and adolescents with obesity, there was no significant difference in the male-to-female ratio between the low- and high-MF groups.

Our study observed that children and adolescents with obesity derive distinct benefits from diet- and exercise-based interventions, regardless of their initial MF. However, some indicators showed less apparent benefits, including a decrease in HDL-C across all four subgroups and no improvement in FBG in either low-MF males or high-MF females. Additionally, we observed that males with an initial high MF experienced greater improvements in body circumference and cardiometabolic indicators than their counterparts with a low initial MF, especially in WC, HC, WHR, TG, TC, and LDL-C. However, this phenomenon was not observed in females. This may be attributed to the similar BFR between females in the high and low MF groups in this study, as well as the significantly higher initial BW and BMI of females in the high MF group than in their low MF counterparts. According to Gerken and Otto et al. [43,44], grip strength, an indicator of MF, can serve as a straightforward and user-friendly measure for predicting weight loss variations. This can contribute to the development of more accurate weight loss strategies for diverse individuals with obesity. Finally, we observed that female subjects did not achieve comparable improvements to their male counterparts at the same MF level. Low MF females exhibited smaller changes in BW, BMI, WHR, TC, HDL-C, and LDL-C compared to low MF males. High MF females exhibited smaller changes in BW, RHR, TG, TC, HDL-C, and LDL-C compared to high MF males. This highlights the importance of paying special attention to changes in anthropometry and cardiometabolic indicators during weight loss in female children.

This study has several limitations. First, the baseline data are inconsistent, as energy expenditure varies in subjects even with the same intervention. Baseline levels of energy intake should be further calibrated to ensure the objectivity of the results. Second, MF was assessed using grip strength. However, factors such as hand posture, joint position, frequency and duration of testing, and expertise of the assessor can influence the accuracy of grip strength measurements, potentially impacting our results. Finally, due to the absence of a control group, caution should be exercised when attributing the findings to the intervention, since other factors may have contributed. Further clinical randomized controlled trials with high quality and extended follow-up durations are essential to obtain more convincing results in the future.

## 5. Conclusions

In conclusion, this retrospective study indicates that at baseline, high MF children and adolescents with obesity are more likely to develop MUO. Males with high MF experienced greater improvements in body circumference and blood lipids from the diet- and exercise-based lifestyle intervention, while this phenomenon was not observed in females. Weight loss effects, such as changes in BFR, BW, and BMI, were not affected by MF differences between individuals of the same sex. However, the benefits of weight loss and blood lipid improvement obtained by males are more evident than those obtained by females when their MF levels are the same. Thus, special attention should be paid to female individuals during weight loss, regardless of their MF level. Early targeted interventions, such as strategies to lower BP and prevent the decline in HDL-C, should be considered in the clinical management of obesity with the objective of improving metabolic health.

## Figures and Tables

**Table 1:** Baseline information of participants.

Variable	Low MF	High MF	Total	*p* Value
N (%)	188 (66.7%)	94 (33.3%)	282 (100%)	/
Age (y)	12.3 ± 2.3	13.5 ± 2.1	12.9 ± 2.3	0.074
Sex (M/F)	100/88	51/43	151/131	0.866
Height (cm)	161.6 ± 11.1	164 ± 10.5	162.4 ± 11	0.049
BW (kg)	80.4 ± 20	84.2 ± 18	81.7 ± 19.4	0.073
BMI (kg/m^2^)	30.3 ± 4.5	31 ± 4.3	30.5 ± 4.5	0.208
BFR (%)	40.4 ± 5.2	39.6 ± 5.2	40.1 ± 5.2	0.213
CC (cm)	98.3 ± 11	100.1 ± 9.6	98.9 ± 10.6	0.261
WC (cm)	96.6 ± 11.8	98.9 ± 10.4	97.4 ± 11.4	0.122
HC (cm)	105.1 ± 10.6	107.4 ± 9.4	105.8 ± 10.3	0.142
WHR	0.92 ± 0.07	0.92 ± 0.06	0.92 ± 0.07	0.903
HDL-C (mmol/L)	1.2 ± 0.2	1.2 ± 0.2	1.2 ± 0.2	0.122
LDL-C (mmol/L)	2.7 ± 0.7	2.8 ± 0.7	2.8 ± 0.7	0.310
TG (mmol/L)	1 ± 0.5	1 ± 0.4	1 ± 0.5	0.615
TC (mmol/L)	4.4 ± 0.9	4.5 ± 0.9	4.4 ± 0.9	0.351
SBP (mmHg)	117.5 ± 15.3	120.7 ± 15	118.6 ± 15.3	0.103
DBP (mmHg)	72.1 ± 12.9	74.6 ± 15.5	72.9 ± 13.9	0.216
FBG (mmol/L)	4.6 ± 0.4	4.6 ± 0.4	4.6 ± 0.4	0.515

Data are presented as mean ± SD. *p* value indicates a comparison of variables between the low MF and high MF groups. MF, muscle fitness; N, number; M/F, male/female; BW, body weight; BMI, body mass index; BFR, body fat ratio; CC, chest circumference; WC, waist circumference; HC, hip circumference; WHR, waist-hip ratio; HDL-C, high-density lipoprotein cholesterol; LDL-C, low-density lipoprotein cholesterol; TG, triglycerides; TC, total cholesterol; SBP, systolic blood pressure; DBP, diastolic blood pressure; FBG, fasting blood glucose.

**Table 2:** Quantity distribution of metabolically healthy indicators in the Low MF and High MF groups before and after intervention.

Variable	Low MF (*n* = 188)				High MF (*n* = 94)			
	MHO		MUO		MHO		MUO	
	Before (%)	After (%)	Before (%)	After (%)	Before (%)	After (%)	Before (%)	After (%)
N	76 (40.4)	47 (25)	112 (59.6)	72 (38.3)	26 (27.7)	17 (18.1)	68 (72.3) *	49 (52.1)
BMI > 95th percentile ^1^	76 (40.4)	68 (36.2)	112 (59.6)	100 (53.2)	26 (27.7)	26 (27.7)	68 (72.3)	64 (68.1)
HDL-C ≤ 1.03 mmol/L	0 (0)	24 (12.8)	45 (23.9)	60 (31.9)	0 (0)	5 (5.3)	31 (33)	42 (44.7) ^##^
TG > 1.7 mmol/L	0 (0)	0 (0)	16 (8.5)	2 (1.1)	0 (0)	0 (0)	6 (6.4)	0 (0)
SBP > 90th percentile ^1^	0 (0)	5 (2.7)	70 (37.2)	21 (11.2)	0 (0)	2 (2.1)	47 (50)	15 (16)
DBP > 90th percentile ^1^	0 (0)	3 (1.6)	54 (28.7)	23 (12.2)	0 (0)	3 (3.2)	34 (36.2)	9 (9.6)
FBG > 5.6 mmol/L	0 (0)	1 (0.5)	2 (1.1)	0 (0)	0 (0)	0 (0)	1 (1.1)	0 (0)

“*” means a comparison of MUO proportions before intervention between low MF and high MF groups, with * *p* < 0.05; “^#^” means a comparison of changes in metabolically healthy indicators after intervention between low MF and high MF groups, with ^##^ *p* < 0.01; ^1^ Age- and sex-matched percentiles, and additional height-matching for blood pressure levels. N, number; MF, Muscle Fitness; BMI, body mass index; HDL-C, high-density lipoprotein cholesterol; TG, triglycerides; SBP, systolic blood pressure; DBP, diastolic blood pressure; FBG, fasting blood glucose.

**Table 3:** Quantity distribution of metabolically healthy indicators in Low MF and High MF groups among different sexes before and after intervention.

Variable			Low MF (*n* = 188)						High MF (*n* = 94)			
	Males		Females		Total		Males		Females		Total	
	Before (%)	After (%)	Before (%)	After (%)	Before (%)	After (%)	Before (%)	After (%)	Before (%)	After (%)	Before (%)	After (%)
BMI > 95th percentile ^1^	100 (53.2)	91 (48.4)	88 (46.8)	77 (41)	188 (100)	168 (89.4)	51 (54.3)	48 (51.1)	43 (45.7)	42 (44.7)	94 (100)	90 (95.7)
HDL-C ≤ 1.03 mmol/L	30 (16)	55 (29.3)	15 (8)	29 (15.4)	45 (23.9)	84 (44.7)	18 (19.1)	30 (31.9)	13 (13.8)	17 (18.1)	31 (33)	47 (50)
TG > 1.7 mmol/L	11 (5.9)	1 (0.5)	5 (2.7)	1 (0.5)	16 (8.5)	2 (1.1)	3 (3.2)	0 (0)	3 (3.2)	0 (0)	6 (6.4)	0 (0)
SBP > 90th percentile ^1^	42 (22.3)	16 (8.5)	28 (14.9)	10 (5.3)	70 (37.2)	26 (13.8)	25 (26.6)	3 (3.2)	22 (23.4)	14 (14.9)	47 (50)	17 (18.1)
DBP > 90th percentile ^1^	29 (15.4)	18 (9.6)	25 (13.3)	8 (4.3)	54 (28.7)	26 (13.8)	15 (16)	5 (5.3)	19 (20.2)	7 (7.4)	34 (36.2)	12 (12.8)
FBG > 5.6 mmol/L	1 (0.5)	0 (0)	1 (0.5)	1 (0.5)	2 (1.1)	1 (0.5)	1 (1.1)	0 (0)	0 (0)	0 (0)	1 (1.1)	0 (0)

^1^ Age- and sex-matched percentiles and additional height-matching for blood pressure levels. N, number; MF, Muscle Fitness; BMI, body mass index; HDL-C, high-density lipoprotein cholesterol; TG, triglycerides; SBP, systolic blood pressure; DBP, diastolic blood pressure; FBG, fasting blood glucose.

**Table 4:** Comparison of changes in indicators between the low MF group and high MF group before and after intervention.

Variable	Low MF				High MF			
	Males (*n* = 100)		Females (*n* = 88)		Males (*n* = 51)		Females (*n* = 43)	
	Before	After	Before	After	Before	After	Before	After
BFR (%)	39.9 ± 5.2	35.3 ± 5.6 ^**ΔΔ**^	41 ± 5.2	36.7 ± 6.4 ^**ΔΔ**^	38 ± 5.6 *	32.7 ± 6.5 ^**ΔΔ**^	41.5 ± 4 ^##^	37.1 ± 5.4 ^**ΔΔ**^
BW (kg)	86.4 ± 21.4	78 ± 19.3 ^**ΔΔ**^	73.6 ± 15.9 ^##^	67.1 ± 15 ^**ΔΔ§§**^	85.7 ± 18.4	77.3 ± 16.8 ^**ΔΔ**^	82.4 ± 17.7 **	75.1 ± 16.4 ^**ΔΔ§**^
BMI (kg/m^2^)	31.6 ± 4.4	28.4 ± 4.1 ^**ΔΔ**^	28.9 ± 4.3 ^##^	26.3 ± 4 ^**ΔΔ§§**^	30.7 ± 4	27.6 ± 3.6 ^**ΔΔ**^	31.4 ± 4.7 **	28.5 ± 4.4 ^**ΔΔ**^
Grip strength (kg)	25.2 ± 9	26.9 ± 9.4 ^**ΔΔ**^	20.6 ± 5.2 ^##^	21.5 ± 6 ^**ΔΔ§**^	32.5 ± 10.7 **	31.3 ± 11.2 ^**Δ**Ϯ^	28.1 ± 7.2 **	28 ± 7.5
Grip strength index	0.29 ± 0.07	0.34 ± 0.07 ^**ΔΔ**^	0.28 ± 0.05	0.32 ± 0.07 ^**ΔΔ§**^	0.38 ± 0.08 **	0.4 ± 0.09 ^**ΔΔ**^	0.34 ± 0.05 **^#^	0.37 ± 0.07 ^**ΔΔ**^
CC (cm)	101 ± 10.6	93.5 ± 9.6 ^**ΔΔ**^	95.4 ± 10.7 ^##^	88.9 ± 9.9 ^**ΔΔ**^	99.8 ± 9.4	91.9 ± 9.5 ^**ΔΔ**^	100.5 ± 9.9 *	94.1 ± 9.2 ^**ΔΔ**^
WC (cm)	101.2 ± 11.3	91.9 ± 10.8 ^**ΔΔ**^	91.5 ± 10.2 ^##^	83.1 ± 9.8 ^**ΔΔ**^	100.4 ± 9.6	88.5 ± 9.7 ^**ΔΔ**ϮϮ^	97 ± 11 **	87.4 ± 10.6 ^**ΔΔ**^
HC (cm)	106.2 ± 10.6	100.5 ± 10.2 ^**ΔΔ**^	103.7 ± 10.6	98 ± 10.2 ^**ΔΔ**^	106.2 ± 9.2	99.1 ± 9 ^**ΔΔ**Ϯ^	108.7 ± 9.6 *	102.7 ± 9.6 ^**ΔΔ**^
WHR (cm)	0.95 ± 0.05	0.91 ± 0.06 ^**ΔΔ**^	0.88 ± 0.07 ^##^	0.85 ± 0.07 ^**ΔΔ§**^	0.95 ± 0.05	0.89 ± 0.05 ^**ΔΔ**Ϯ^	0.89 ± 0.06 ^##^	0.85 ± 0.06 ^**ΔΔ**^
SBP (mmHg)	121.8 ± 15.6	110.6 ± 13.2 ^**ΔΔ**^	112.5 ± 13.4 ^##^	103.9 ± 10.8 ^**ΔΔ**^	122.1 ± 16.1	109.3 ± 10.7 ^**ΔΔ**^	119.1 ± 13.7 *	110.7 ± 15.7 ^**ΔΔ**^
DBP (mmHg)	73.3 ± 14.8	65.9 ± 10.9 ^**ΔΔ**^	70.7 ± 10.4	62.8 ± 10.3 ^**ΔΔ**^	74.5 ± 16.8	63.9 ± 12 ^**ΔΔ**^	74.7 ± 14	65.2 ± 12 ^**ΔΔ**^
RHR (beats/min)	92.2 ± 12	81.8 ± 12.9 ^**ΔΔ**^	90.5 ± 13.2	80.7 ± 12.1 ^**ΔΔ**^	91.6 ± 13.2	78.3 ± 9.9 ^**ΔΔ**^	92.7 ± 13.5	85.7 ± 15 ^**ΔΔ§§**^
FBG (mmol/L)	4.6 ± 0.4	4.5 ± 0.3	4.6 ± 0.5	4.4 ± 0.5 ^**ΔΔ**^	4.6 ± 0.4	4.4 ± 0.4 ^**ΔΔ**^	4.6 ± 0.3	4.5 ± 0.3
TG (mmol/L)	1 ± 0.6	0.7 ± 0.3 ^**ΔΔ**^	0.9 ± 0.5	0.8 ± 0.3 ^**ΔΔ**^	1 ± 0.5	0.7 ± 0.2 ^**ΔΔ**Ϯ^	0.9 ± 0.4	0.8 ± 0.2 ^**Δ§§**^
TC (mmol/L)	4.5 ± 1	3.5 ± 0.6 ^**ΔΔ**^	4.4 ± 0.8	3.6 ± 0.7 ^**ΔΔ§§**^	4.7 ± 0.9	3.3 ± 0.4 ^**ΔΔ**ϮϮ^	4.4 ± 0.8	3.7 ± 0.6 ^**ΔΔ§§**^
HDL-C (mmol/L)	1.2 ± 0.3	1.1 ± 0.2 ^**ΔΔ**^	1.2 ± 0.2	1.2 ± 0.2 ^**ΔΔ§§**^	1.2 ± 0.3	1 ± 0.2 ^**ΔΔ**^	1.2 ± 0.2	1.1 ± 0.3 ^**Δ§§**^
LDL-C (mmol/L)	2.8 ± 0.7	2 ± 0.5 ^**ΔΔ**^	2.7 ± 0.7	2 ± 0.5 ^**ΔΔ§**^	2.9 ± 0.7	1.9 ± 0.4 ^**ΔΔ**Ϯ^	2.7 ± 0.6	2.1 ± 0.5 ^**ΔΔ§§**^

“*” means a baseline comparison within the same sex group at different MF levels, with * *p* < 0.05 and ** *p* < 0.01; “^#^” means a baseline comparison within different sex groups at the same MF level, with ^#^ *p* < 0.05 and ^##^ *p* < 0.01; “^Δ^” means a comparison between before and after the intervention within the same subgroup under the same conditions of MF level and sex, with ^Δ^ *p* < 0.05 and ^ΔΔ^ *p* < 0.01; “^Ϯ^” means a comparison between the change levels after the intervention within the same sex group at different MF levels, with ^Ϯ^ *p* < 0.05 and ^ϮϮ^ *p* < 0.01; “^§^” means a comparison between the change levels after the intervention within different sex groups at the same MF level, with ^§^ *p* < 0.05 and ^§§^ *p* < 0.01. MF, muscle fitness; BFR, body fat ratio; BW, body weight; BMI, body mass index; CC, chest circumference; WC, waist circumference; HC, hip circumference; WHR, waist-hip ratio; SBP, systolic blood pressure; DBP, diastolic blood pressure; RHR, resting heart rate; FBG, fasting blood glucose; TG, triglycerides; TC, total cholesterol; HDL-C, high-density lipoprotein cholesterol; LDL-C, low-density lipoprotein cholesterol.

## Data Availability

The datasets used and/or analyzed during the current study are available from the corresponding author upon reasonable request.
